# A fuzzy-oscillatory model of medial prefrontal cortex control function in spatial memory retrieval in human navigation function

**DOI:** 10.3389/fnsys.2022.972985

**Published:** 2022-10-24

**Authors:** Maryam Moghadam, Farzad Towhidkhah, Shahriar Gharibzadeh

**Affiliations:** ^1^Department of Biomedical Engineering, Amirkabir University of Technology (Tehran Polytechnic), Tehran, Iran; ^2^Cognitive Rehabilitation Clinic, Institute for Cognitive and Brain Sciences, Shahid Beheshti University, Tehran, Iran

**Keywords:** navigation, hippocampus, medial prefrontal cortex, interaction, Van-der-pol oscillator, rule-based fuzzy system, retrieval control

## Abstract

Navigation can be broadly defined as the process of moving from an origin to a destination through path-planning. Previous research has shown that navigation is mainly related to the function of the medial temporal lobe (MTL), including the hippocampus (HPC), and medial prefrontal cortex (mPFC), which controls retrieval of the spatial memories from this region. In this study, we suggested a cognitive and computational model of human navigation with a focus on mutual interactions between the hippocampus (HPC) and the mPFC using the concept of synchrony. The Van-der-pol oscillator was used to model the synchronous process of receiving and processing “what stream” information. A fuzzy lookup table system was applied for modeling the controlling function of the mPFC in retrieving spatial information from the HPC. The effect of attention level was also included and simulated. The performance of the model was evaluated using information reported in previous experimental research. Due to the inherent stability of the proposed fuzzy-oscillatory model, it is less sensitive to the exact values of the initial conditions, and therefore, it is shown that it is consistent with the actual human performance in real environments. Analyzing the proposed cognitive and fuzzy-oscillatory computational model demonstrates that the model is able to reproduce certain cognitive and functional disturbances in navigation in related diseases such as Alzheimer’s disease (AD). We have shown that an increase in the bifurcation parameter of the Van-der-pol equation represents an increase in the low-frequency spectral power density and a decrease in the high-frequency spectral power as occurs in AD due to an increase in the amyloid plaques in the brain. These changes in the frequency characteristics of neuronal activity, in turn, lead to impaired recall and retrieval of landmarks information and learned routes upon encountering them. As a result, and because of the wrong frequency code being transmitted, the relevant set of rules in the mPFC is not activated, or another unrelated set will be activated, which leads to forgetfulness and erroneous decisions in routing and eventually losing the route in Alzheimer’s patients.

## Introduction

Navigation is the process of moving from a current location (origin) to a predetermined goal on a path resulting from the process of planning. Indeed, navigation is a cognitive process that enables the person to remember different places and spatial relations between objects and find their path toward the goal. In recent years, navigation has been the subject of much research especially since it is an essential cognitive function for all animals and humans, and because of the increasing number of occurrences of navigational difficulties in dementia-related diseases such as Alzheimer’s disease. In fact, navigational difficulties are common in patients with different types of dementia (not only AD) and more than 60% of persons with dementia will wander. This clearly shows the importance of the issue.

Due to the multi-faceted nature of human navigation and routing, there are a multitude of approaches in related research, e.g., in cognitive and computational modeling of its different aspects. Some deal with this issue at the cellular (microscopic) level, some at the mesoscopic level, and some at the macroscopic and functional level (Madl et al., 2015).

Some studies only investigate the performance of one brain area during the relevant task (Nyberg et al., 2022), some others investigate, study, and model the interaction of several brain areas during the relevant task like (Chersi and Burgess, 2015). Also, some studies include the process of learning and finding the optimal path (with different approaches e.g., the shortest time or the shortest path, etc.), or planning in navigation, and use Markov’s and Bayesian decision modeling to update map-learning based on observed evidence (Kaplan and Friston, 2018). The other approach is to only investigate how the learned maps are recalled and the correct route is chosen based on retrieved memory.

Due to the inherent complexities of the navigation problem and the simultaneous activity of many brain areas during this cognitive task, and because in various studies only a part of the related brain areas and certain aspects of the navigation process have been studied and modeled; far more studies are needed in this field to provide a more comprehensive cognitive and computational model of navigation.

In this study, our aim is to provide a cognitive and computational model of human navigation with a stronger focus on mutual interactions between the hippocampus (HPC) and the mPFC (as spatial memory retrieval controller) using the concept of synchrony. The assumption of the problem is that learning has been done. Also, in this study, considering the functional nature of different brain regions, a computational model with greater similarity to the actual human performance in retrieving information during navigation has been presented as compared to previous models. With this explanation, we will continue to explain the topics and studies in this framework.

The process of navigation depends on two types of representations: egocentric (self-to-object) and allocentric (object-to-object). Since egocentric information and path integration are not sufficient, the allocentric representation is necessary for navigation towards a destination that is initially not observable. Evidence exists regarding the role of the parietal lobe and PFC in providing egocentric representations and short-term memory (STM), whereas medial temporal lobe (MTL) provides allocentric representation and long-term memory (LTM; Hartley et al., 2004). The allocentric representation is in fact, a result of processing and storage of information by different HPC cells.

The HPC is a c-shaped cortical structure that forms an important part of the MTL. There is a hippocampal segment in each hemisphere of the brain. The term “hippocampal formation” often refers to the HPC along with its related structures including the subiculum and entorhinal cortex (EC). The EC is located behind the HPC and connects it to the cortical areas and provides an important input to the HPC. The HPC consists of different cell structures like pyramidal cells including Cornu Ammonis1–3 (CA1, CA2, CA3) which are also called place cells, and granule cells including the dentate gyrus (DG), which have different tasks and are arranged along the longitudinal axis of the hippocampus in an orderly manner. According to studies, the EC, DG, and CA1–3 areas and the synaptic circuits between these areas play a decisive role in learning and retrieval. With the discovery of the types of HPC formation cells and their functions, new mechanisms of routing and navigation were understood. To read more about the anatomical details of the hippocampus, the interested reader can refer to (Anand and Dhikav, 2012).

It is often stated that spatial memories are stored as cognitive maps (Moscovitch et al., 2005), and such maps are used in the process of path planning. According to Tolman’s study, the cognitive map is in fact a unique representation of the spatial environment that the brain creates and employs for supporting the memory and guiding further steps (Tolman, 1948). The process of navigation based on cognitive maps is mostly related to the function of the MTL including the HPC (Burgess et al., 2002), and the cognitive map is stored and presented by different types of HPC formation cells including place cells, grid cells, boundary cells, and head direction cells (O’Keefe and Dostrovsky, 1971; Eichenbaum et al., 1999; Fyhn et al., 2004; Moser et al., 2015; Epstein et al., 2017; Nyberg et al., 2022).

The HPC and mPFC regions strongly contribute to the encoding and retrival of episodic memories (Jin and Maren, 2015). It has been shown through spatial working memory tasks that damage to the PFC impairs the spatial firing of the HPC place cells (Jin and Maren, 2015). The HPC plays a vital role in long-term memory, and it has also been reported to be involved in short-term memory, where certain modes of spatial processing are required even for very brief periods (Hartley et al., 2007). Lesion and imaging studies show that episodic memory retaining and retrieval are always dependent on the HPC, whereas semantic memories possibly benefit from the HPC while still being able to do without it. Recent evidence indicated that new events are learned in the context of existing memories. In the HPC and mPFC, related memories are represented by integrated codes. These codes constitute the basis for spatial, temporal, and conceptual maps that resulted from every experience (Morton et al., 2017).

Decision-making is yet another principle issue in routing (Patai and Spiers, 2021) in order to choose the correct route at crossroads. When we make a decision, we often need to consider the available options in order to choose the most appropriate one. This process requires an evaluation of the pros and cons of previously available options, and this, in turn, depends on the memories of previous actions and their corresponding outcomes. It has been reported that HPC and PFC are needed for encoding and retrieval of information and decision making (Yu and Frank, 2015; Nyberg et al., 2022). These regions interact through oscillatory synchronization in the theta frequency band (Place et al., 2016). Theta band activity increases with increased working memory load (Jensen and Tesche, 2002; Kaplan et al., 2017).

In 2013, Preston and Eichenbaum (2013) showed that mPFC and medial entorhinal cortex (MEC) play distinct roles in retrieving representations of context-based memories stored in the HPC. The mPFC provides contextual control over the retrieval of object location memories using strict rules, whereas the MEC provides the required information for HPC to map the spatial context, including where essential events occurred (Preston and Eichenbaum, 2013). Later in 2016, they focused on the dynamics of interaction between the HPC and PFC in rats during the use of spatial context for guiding the retrieval of object memory. Analysis of functional information in this study has shown that while entering the context (i.e., the place where the objects are located), the contextual information stream would be from the HPC towards the PFC. However, upon beginning to focus on the objects themselves and sampling thereof, the information stream is reversed. This finding lends support to the fact that the PFC controls the retrieval of HPC memory representations corresponding to context (Place et al., 2016). So, it seems that HPC supports mPFC with bottom-up information and mPFC in a top-down manner affects prospective spatial representations (Brown et al., 2016; Nyberg et al., 2022).

In 2015, Chersi and Burgess (2015) paid attention to the collaboration between the HPC and striatum. Using the previous findings, they introduced a simplified cognitive architecture that included: 1-reinforcement learning by the striatum on the basis of representations of sensory and practical states, 2-implicit connections between sensory information and representations of allocentric conditions in the HPC, and 3-comparing and judging the outputs of both systems based on confidence or uncertainty in the mPFC (Chersi and Burgess, 2015). Despite alleviating the limitations of previous models by taking the role of striatum into account, the mutual relations between the HPC and PFC were not considered in this model. In addition, the role of the PFC is limited to solely judging between two inputs from the striatum and the HPC.

Available data, demonstrate that the HPC-mPFC interaction during the encoding and retrieval of the episodic memory is dynamic, and these regions are strongly involved in encoding and retrieval of episodic memories (Jin and Maren, 2015). Also, this mutual interaction supports content-dependent memory encoding and retrieval (Preston and Eichenbaum, 2013). There is abundant evidence that in the HPC-mPFC mutual information stream, events triggering the PFC control over the HPC memory retrieval originate from the ventral/anterior HPC. Information from these regions is directly sent to the mPFC, and the mPFC affects the retrieval of the specific object representation through its strong connections to the perirhinal (PRC) and lateral entorhinal cortex (LEC) cortical regions (Preston and Eichenbaum, 2013; Zangbar et al., 2020).

The HPC is viewed as a constructor and retriever of specific memories, whereas the PFC integrates features of related memories, which constitutes the *context*, from a set of continuous experiences, such as a common place in which several events have occurred or a common set of implementing rule tasks that manage multi-memory decisions. In this way, whenever the PFC points to a specific context or content, it subsequently biases and activates context or content-related memories in the HPC and other brain regions and “turns off” non-related memories. In fact, the mPFC employs these contextual representations to control the retrieval of detailed memories in the HPC. Studies show that the neuronal populations in the mPFC are fired selectively in behavioral contexts. These neuronal activity patterns in the mPFC are predictors for the switching between the recall of place and response strategies in the spatial memory domain. When the mPFC is inactivated, dorsal HPC neurons non-selectively retrieve both related and non-related objects’ memory representations. These findings show that the HPC is able to retrieve memories even in the absence of input from the mPFC, and the actual role of the mPFC is to select the appropriate memory for a specific context or content. Based on this scenario, in addition to environmental landmarks that define a context, events occurring in that context are processed by the anterior/ventral HPC (Preston and Eichenbaum, 2013). Through mapping and direct connection, the present location and local trajectory information (Jin and Maren, 2015) are sent to the mPFC, where neural ensembles create a set of distinct contextual rules during the learning process. Consequently, after the learning process, when the person is placed in the same context, and the processed sensory information arrives at the mPFC directly from the anterior/ventral HPC, mPFC applies rules corresponding to the context. This is performed to employ appropriate representations (with regard to the context) in the dorsal/posterior HPC while suppressing context-irrelevant memories (Preston and Eichenbaum, 2013) and making the final decision based on the retrospective and prospective information received from HPC (Nyberg et al., 2022). These concepts constitue the basis for the novelties of this article.

This article is a novel attempt to address some of the challenges and shortcomings in the current understanding and modeling of navigation. Despite advances in this field, there is still much ambiguity surrounding important aspects of navigation. These include how mammal brains represent goal locations; or how goal-related representations are transferred from the Hippocampal formation to other regions of the brain. Goal identification codes depend on the type and stage of navigation. The importance of clarifying the navigational strategies and demands from which the goal codes originate has thus been underlined by previous reports (Nyberg et al., 2022).

In this article, we present a step towards clarifying the process of navigation and what happens in the retrieval of information relating to the location and nature of the goal and subgoals. To overcome the above-mentioned ambiguities, a new cognitive and computational model of navigation with more focus on the HPC-mPFC interaction and the control function of the mPFC in memory retrieval during navigation is proposed.

Some novelties of this study in comparison with previous navigation cognitive and computational models are as bellow:

▪ Inclusion of the mutual interaction between the HPC and mPFC in the cognitive and computational models.
▪ Inclusion of the short-term memory unit and as a consequence, the spatial working memory.
▪ Computational modeling of the mPFC as the controller of cognitive map memory retrieval.
▪ Providing suggestions for modeling the *what* and *where* information streams during navigation.
▪ Modeling based on the concept of synchrony, and a new analysis of the cause of forgetfulness and losing the path in people with Alzheimer’s by analyzing changes in parameters.
▪ Inclusion of the attention level parameter in the navigation model.

## Background

In the following, we explain our proposed cognitive model. The cognitive and computational model scenario is based on the synchrony mechanism in the brain regions’ interactions corresponding to cognitive functions. Thus, we first provide a brief explanation about the synchrony mechanism in brain interactions.

### Theory of brain communication based on synchronous mechanism

One of the central issues in cognitive neuroscience is the question of which processes provide for communications within and between neural networks in the brain. The basic hypothesis is that phase synchrony plays an important role in process binding, and this can be one special type of large-scale communication within the brain. Studies have shown that synchronous theta band activity between the HPC and mPFC is necessary for spatial working memory and remote memory recall (Wirt and Hyman, 2017). Findings show that theta-alpha phase pairing reflects the control processes in the extensive memory system, and theta phase synchronization provides controlled access to episodic memory (Klimesch et al., 2010). Theta band activity specifically increases during the observation of landmarks. Similarly, increasing and amplifying theta power during navigation toward the goal compared to navigation without a goal demonstrates the role of theta oscillations in retrieving spatial memory and path planning (Zangbar et al., 2020; Nyberg et al., 2022). Such an increase in theta power has also been observed in navigation over longer paths which is due to the need to retrieve more spatial information and longer path planning. The higher theta band power in familiar environments can be justified by the fact that more spatial information is available that can be used and retrieved to guide motion (Herweg and Kahana, 2018). Theta signal has been observed both in the encoding and retrieval phases. The nature of the transferred information is still unknown (Wirt and Hyman, 2017), and only several hypotheses have been suggested based on observations from laboratory recordings. Considering the complicated nature of brain functions, we have presented a relatively inclusive cognitive model by comprehensively reviewing highly cited research reports in Moghadam et al. (2021). The proposed model is based on the concept of synchrony in both encoding and retrieval.

### A cognitive model of navigation towards the initially unobservable destination

**Figure 1:**
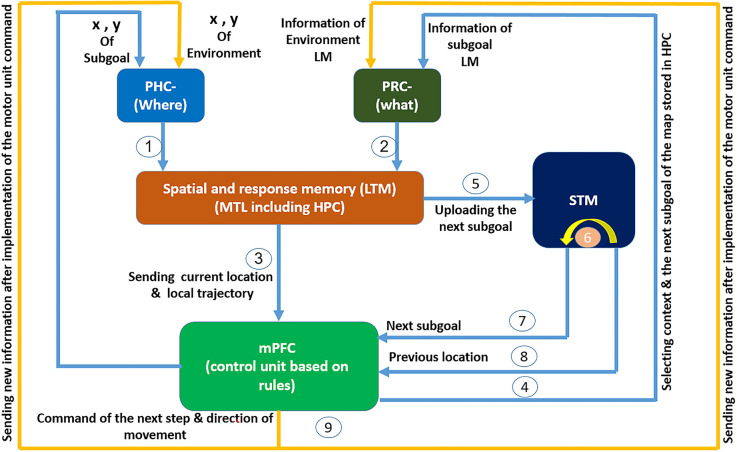
The cognitive model of navigation focused on mPFC role in spatial memory retrieval control: spatial information regarding the agent’s location and the nature and location of objects arrives at the PHC and PRC through the *what* and *where* streams. After preliminary processing, the information reaches the spatial and response memory structures through information pathways 1 and 2. These structures are where place cells, grid cells, and head direction cells reside. subsequently and after integration and processing of spatial information in this region, information regarding the agent’s current place and the local trajectory enters the PFC through path number 3, where the PFC acts as a control unit responsible for controlling memory retrieval. In the PFC, spatial information is matched against the rules sets that have been formed in mPFC during learning. Then, based on the input information, the context and address code for the next expected landmark are fed back by the mPFC to the HPC where the cognitive map is stored, through path number 4 and the PRC. In the HPC this code is matched against the cognitive map and the next expected landmark is loaded into the memory. This loaded data is compared with the input information from the environment through the what stream. If a mismatch is observed, a “wrong path” message follows in order for the agent to correct its choice. If the environmental information matches the cognitive map, the next expected landmark information (which itself results from the control unit’s prediction and recall from the cognitive map) including its characteristics and location, as well as the agent’s current location are sent to the next subgoal buffer in the short-term memory through path number 5 and its previous stored information shifts to the previous spatial buffer through path number 6. This information is made available to the spatial working memory and the control unit through paths number 7 and 8, such that an appendage of such information to the response memories will transmit the next step command and its direction to the motor units. After that movement command has been executed, the new agent’s location and environmental landmarks enter the sensory inputs through path number 9 and this cycle continues until the goal is reached.

Our proposed model is based on an extensive survey of existing cognitive, behavioral, and biological literature. This model was presented regarding the function of the brain’s active regions involved in cognitive-map-based navigation and their interrelations. Figure 1 shows the model in a step-by-step and continuous manner from the input of sensory information to the final processing and execution in a complete performance loop.

As mentioned, the complete set of activities by place cells, grid cells, boundary cells, velocity, and head direction cells constitute the cognitive map during the stage of learning. The next step of the cognitive map toward the goal is retrieved by updating current information from the head direction cells and locations of the agent and the landmarks under the control of the mPFC. This information is compared to environmental information to assess the correctness of chosen steps towards the goal.

**Figure 2:**
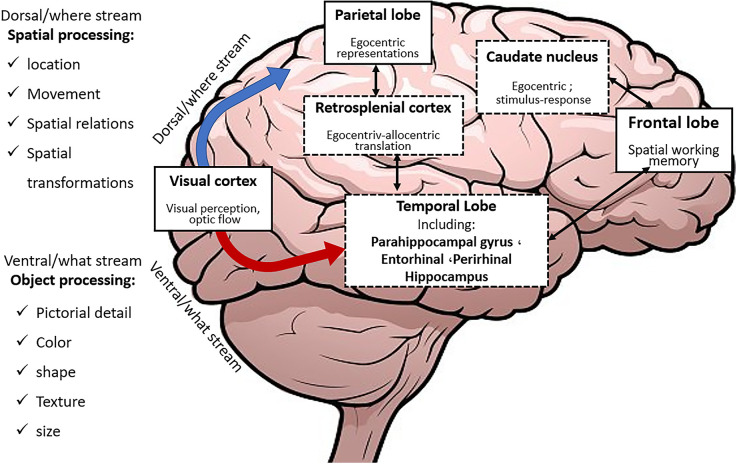
The What and Where streams within the brain.

Percepted environmental information about objects and events is initially processed in specific brain regions of various sensory modalities and constitutes the *what* and the *where streams* (Figure 2).

The what stream is directed to the PRC and LEC while the where stream is directed towards the retrosplenial cortex (RSC), parahippocampal cortex (PHC), and MEC after being processed in the posterior parietal region. Subsequently, these two streams converge in the HPC. Then the PHC and RSC provide the fundamental inputs which allow the stored cognitive map within the HPC to anchor to fixed environmental landmarks. Landmark anchoring helps faster and more exact learning and retrieval.

In the dorsal (animals)/posterior (humans) HPC, neuronal populations store particular objects and places that have occurred in a specific context. On the contrary, neuronal ensembles in the ventral (animals)/anterior (humans) regions of the HPC connect events within the context and hence create distinctions between different contexts (Preston and Eichenbaum, 2013).

As described earlier in this section, the PFC is a vital structure that supports the working memory and executive functions, and its activation is generally observed during the entire navigation task (Zangbar et al., 2020); especially when the person has to deviate from the trajectory line in order to reach the goal (Lithfous et al., 2013). It can therefore be concluded that the stored rules within the mPFC are formed at the main landmarks, where turning and path changing occur. Upon reaching these landmarks, the control unit issues the proportionate command to continue the correct path. This is the main idea in considering the *information of the key points* (landmarks) in (1; Moghadam et al., 2021).

The information regarding the temporal sequence of landmark encounters during navigation are also required for the formation of associated links between subsequent landmarks and associative behavioral responses. Studies show that during the wayfinding task, which requires recall of the sequential order of the landmarks, the medial frontal gyrus experiences higher activity. Also, the frontal cortex has been found to play a fundamental role in storing the temporal order of landmarks (Lithfous et al., 2013).

According to recent research, the context (Wirt and Hyman, 2017) and goal locations are also stored in the mPFC (Wirt and Hyman, 2017; Ito, 2018). This information is employed to plan successive goal locations (Ito, 2018). It has also been shown that following mPFC damage, navigation from a new beginning point towards a specific goal is impaired. This shows, in turn, that the correct function of mPFC is necessary for navigation based on the cognitive map from any start point to arbitrary goals and enables flexible path planning towards a specific goal. Based on these findings, the order of encounter is stored in the rules (which are formed during the learning process) in the PFC (mPFC control block in Figure 1) in the form of (1):


(1) $$\left(LM-Code\;(here)\;LM-Code\;(Goal)\text{ Attention level }LM-Code\;(next\;step)\right)$$

According to (1), for each step, considering the current location of the agent [*LM-Code(here)*], and the landmarks in its field of view, and in accordance with the final goal [*LM-Code(Goal)*], the appropriate code relevant to the next step and subgoal [*LM-Code(next step)*] is stored as a consequent of the related rule within the PFC.

The whole set of these steps from the start to the goal are stored in PFC as a set of specific rules for each context and path. Thus, in each step, regarding the environmental information sent from HPC to mPFC and based on the person’s attention level, the code for the next expected landmark and next step is extracted from the related stored rule and sent to HPC. The stored memory corresponding to that code is then retrieved from a related cognitive map stored in the HPC place cells.

Previous studies have shown that in dorsal (animals)/posterior (humans) HPC, neuronal ensembles store specific objects and places where they are located in context. It has been shown that place cells fire according to the *distance* to the upcoming goal, which demonstrates the representation of the expected upcoming place by place cells. HPC place cells not only show the agent’s current location but also show the past or future path by changing the firing rate (McNaughton et al., 2006). Therefore place cells can represent instantaneous, previous, and upcoming locations as well as trajectories (Lithfous et al., 2013). Consequently, the cognitive map stored within the HPC is inclusive of landmark environmental locations and also their relations and distances from the navigator’s viewpoint (Edvardsen et al., 2020). Building upon these findings, and in accordance with Moghadam et al., 2021, the cognitive map stored in the long-term memory is computationally introduced by a matrix as shown in (2).


(2) $$\left(X-LM\;(next\;step)\;Y-LM\;(next\;step)\;W-LM\;(next\;step)\;X-motor\;Y-motor\right)$$

Here, *X-LM* and *Y-LM* are the coordinates of the next landmark, *W-LM* shows the oscillation frequency coding the nature of the next expected landmark, and *X*-motor and *Y*-motor show the current location of the agent. All relations and distances, either egocentric or allocentric, are obtained using this information in the computational neural centers of the brain and are used instantaneously to understand space and motion commands. Therefore, (2) shows the stored cognitive map and the spatial information processing in the spatial and response memory block in Figure 1.

**Figure 3:**
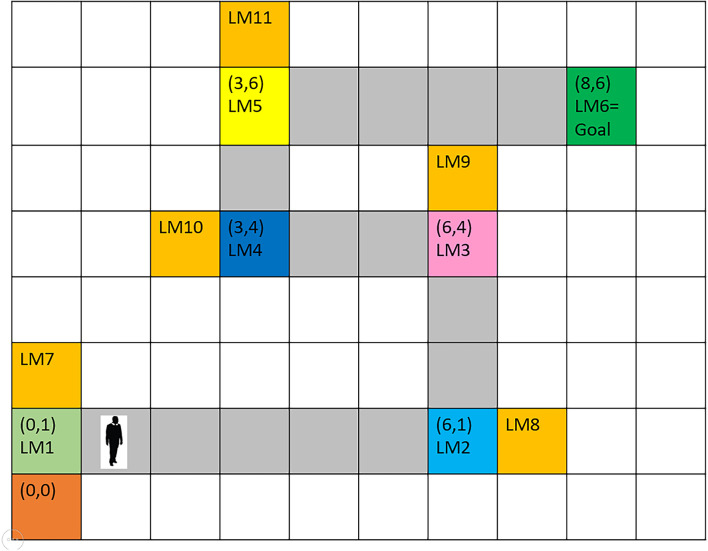
The assumed route for agent’s navigation. Six landmarks are considered from the origin to the destination. It is assumed that at each landmark position, the next landmark is visible and that the landmark positions are the turning points of the route. Numbers in parentheses indicate coordinates with reference to the start point. Gray-colored squares between landmarks depict the correct path. Orange-colored squares indicate wrong paths, which the agent will enter by mistake, in case of low attention to turning points (landmarks).

The mPFC, which is known as a vital region for action planning and decision making, controls which information shall be retrieved at any time and guides the changes in the rate of path-dependent frequency (McNaughton et al., 2006; Ito, 2018). Once the transmitted next-expected-landmark code from the mPFC is matched with the cognitive map, the retrieved information is compared against environmentally obtained information arriving from the what stream. If they disagree because of the agent’s improper path selection, contradiction results in a message of wrong path, so that the agent starts correcting its path and continues navigating towards the goal. If the environmental information agrees with the next expected landmark (predicted by the control unit and retrieved from the cognitive map), information regarding the next landmark, including its location and characteristics and also the current location of the person, is loaded into the short-term memory, i.e., the buffer relevant to the next subgoal, shifting the prior information to a previous buffer. This updated information is now available to the spatial working memory and the control unit, ready to be attached to the response memory, thereby sending the next step command and its direction to the motor unit. Once the agent has executed the movement command, new information regarding the new location of the agent and environmental landmarks are entered *via* the sensory inputs, and this cycle continues until the goal is reached. Figure 3 shows a sample cognitive map used in this study.

The performance of the model was evaluated using information reported in previous experimental research.

Using the mentioned scenario, which has been provided from the aggregation and integration of information reported in previous experimental research, in this article, the HPC-mPFC interaction and the mPFC control function during cognitive map retrieval for navigation are modeled.

## Materials and Methods

In the following, we first explain and implement the proposed computational model, and then the modeling results will be presented.

Following the background given in the introduction, it is understood that the whole set of activities and encodings by HPC cells and other related regions in the MTL, constitute the cognitive map following the stage of learning. Afterward, during navigation on the learned route, information regarding the direction and coordinates of the agent and landmarks is instantaneously updated; the next steps towards the goal are retrieved from the cognitive map under the control of the mPFC and used as expected data to be compared with environmental data and decide about the correctness of the person’s routing options. As mentioned, the perceptual information regarding objects and events are first processed in the particular brain areas of various sensory modalities, which constitute what and where streams. In the following, we provide suggestions for modeling the information flow in these two streams during navigation, and we have explained some of the simulation results.

### Modeling *where* stream function during navigation

In this article, a network of polar neurons is used to model where stream function of the parietal in human brain during navigation. The network includes 360 polar neurons, each equivalent to a specific coordinate in the egocentric system. For computational modeling, a discretization of the space is assumed with 36 divisions in azimuth (10 degrees resolution) and 10 divisions in the radial coordinate. Equation (3) denotes polar neurons:


(3) $$R_{i}^{PW}=e^{-\left(\frac{\theta_{i}^{e}-\theta^{e}}{\sigma_{\theta }}\right)}e^{-\left(\frac{r_{i}-r}{\sigma_{r}}\right)}$$

**Figure 4:**
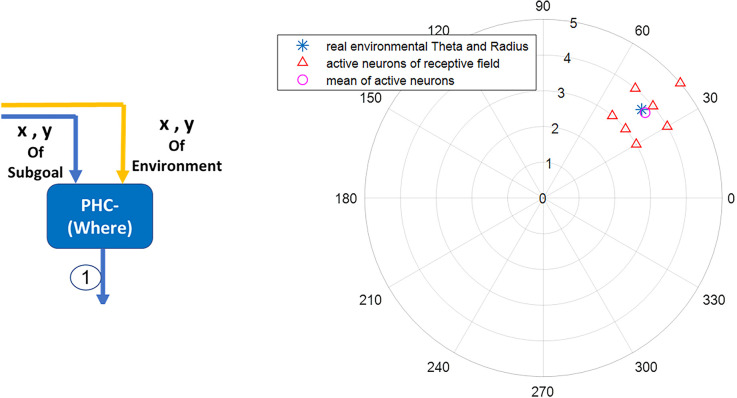
The blue star denotes the landmark’s real coordinates relative to the agent’s body axis. The red triangles denote the activated neurons in the parietal region responsible for the acquisition and processing of egocentric information. The average activity of polar neurons (the pink circle) represents with negligible error the perceived coordinates information of the blue star (the real landmark coordinates) within the environment.

In this equation, (*r*_i_,$\theta_{i}^{e}$) are the egocentric coordinates (radial distance and angle) of the neuron corresponding to the environmental landmark. Also, $\sigma_{r}^{2}$ and $\sigma_{\theta }^{2}$ are considered to be 0.06 and 0.002, respectively. Figure 4 shows simulation results.

### Modeling *what* stream function during navigation using Adaptive Resonance Theory (ART)

According to the adaptive resonance theory (ART) first proposed by Carpenter and Grassberg in a discussion of pattern recognition, sensory information is synchronized and resonated with similar information in memory, thus that information is recognized. If sensory information does not resonate with any of the items in the memory, a learning process begins (Carpenter and Grossberg, 2010).

The presented sensory information tries to influence the brain’s processing resources at different levels. In bottom-up visual and auditory processing centers, there are different pools of neurons, each of which responds to one of the stimulus properties. Thus, it can be said that sets of these pools of neurons in bottom-up processing centers respond individually to route’s landmarks during navigation. Accordingly, in the hypothetical map of this article shown in Figure 3, we have considered six (in a selective manner) Van-der-pol oscillators corresponding to the six main landmarks (LM1–LM6). Thus, other landmarks not already stored in the map of the selected route for navigation to the target will be considered as non-target or distractors.

Each property of a landmark can be equated to, and modeled with a frequency, but for simplification in the computational model, the landmarks are coded with the dominant frequency of the spectrum (the main harmonic). It is also assumed that learning has already been done and the information is stored in the form of a cognitive map.

The reasons for choosing the Van-der-pol equation are:

1- Among the various oscillators in the literature, Van-der-pol oscillator has been one of the oscillators used to model neuronal oscillations.
2- In the Van-der-pol equation, the finer details of the chemical and electrical activities of the various components of the neuronal ensemble are omitted, and neuronal oscillations are modeled with a global view.

Therefore, the use of a Van-der-pol model compared to other oscillator models used to model neuronal activity, keeps the proposed general model from becoming too complex. However, the use of more accurate and complex models as needed may add more richness to the model.


(4) $$\left(\begin{array}{c} {\dot{X}}_{l}=\left(\lambda_{l}-Y_{l}^{2}\right)X_{l}-P_{l}^{2}Y\\ {\dot{Y}}_{l}=X_{l} \end{array}\right)$$

In the Van-der-pol oscillator in Equation (4), *X* and *Y* are the state variable and output of each neuronal ensemble, respectively. When λ is less than zero, there is no oscillation in the ensemble behavior; When the value of λ is between zero and one, the neuronal ensemble will oscillate with frequency P and amplitude 2$\sqrt{\lambda }$ (Balanov et al., 2008).

According to Equation (4), it can be seen that the Van-der-pol oscillator is an inherent oscillator and has no input. In this study, as in (Baghdadi et al., 2018), we used the Van-der-pol oscillator as an ensemble of neurons that respond to input signals. Therefore, considering each landmark as an input excitation in the form of a sine wave with amplitude *A_s_* as excitation (event) intensity and frequency *ω_s_* as a representation of excitation properties, according to theories (Kumar et al., 2010; Onken et al., 2014) based on the frequency coding of information in the brain, we used the Van-der-pol equation with the input of Equation (5). In this relation, *B_l_* is the coupling coefficient between the incoming oscillating input and the neural units.


(5) $$stimulus=B_{l}\times A_{s}\sin\;(\omega_{s}t)$$

According to this model, the receiving field is actually the oscillation frequency of the neuronal ensemble, and in the processing stages, neurons whose oscillation frequency is close to the coded frequency of the input stimulus (the same as the landmarks of the navigation route) show a stronger response than other neurons (Fritz et al., 2007).

**Figure 5:**
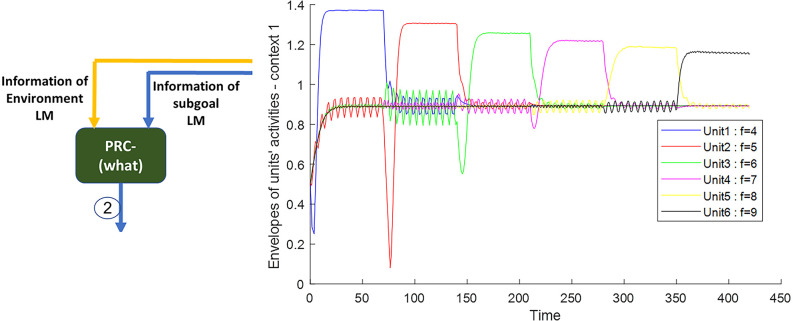
The outputs of the Van-der-pol neuron model which is used in this article to model neuronal ensembles in the PRC region. The simulation results are shown as neuronal units’ responses to input excitations (landmarks 1–6), in the order of encountering based on the route of related context. Right column: amplitude envelop for neuronal units’ responses to input excitations for context 1. The parameters for the Van-der-pol model in this simulation are *λ* = 0.2, *B*_1,‥.,6_ = 1.5, *A*_s_ = 1, *P*_1,‥.,6_ = 4,5,6,7,8,9, *ω*_s_1,‥.,6__ = 4,5,6,7,8,9.

As can be seen from the above description and the modeling results in Figure 5, when the agent reaches any of the 1–6 landmarks specified in Figure 3 (route map), the corresponding unit is activated by the activity function of Equation (6). And the field activity of other units is below the threshold.


(6) $$\left(\begin{array}{c} {\dot{X}}_{l}=\left(\lambda_{l}-Y_{l}^{2}\right)X_{l}-P_{l}^{2}Y+stimulus\\ {\dot{Y}}_{l}=X_{l} \end{array}\right)$$

**Figure 6:**
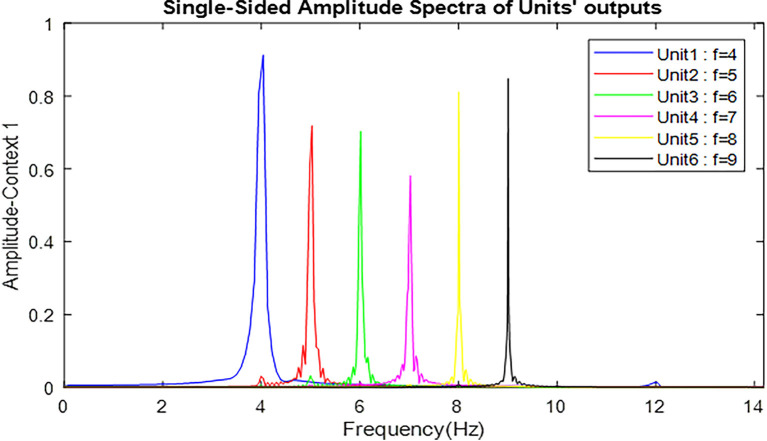
Frequency spectrum of neuronal units’ responses to landmarks of context 1. Frequencies 4–9 in this figure are equivalent to main landmarks 1–6 in Figure 3. The parameters for the Van-der-pol model in this simulation are *λ* = 0.2, *B*_1,‥.,6_ = 1.5, *A*_s_ = 1, *P*_1,‥.,6_ = 4,5,6,7,8,9, *ω*_s_1,‥.,6__ = 4,5,6,7,8,9.

The frequency activity of neuronal ensembles, in this case, is also shown in Figure 6. The difference in the amplitude of the units activity in Figure 5 is due to the effect of the numerical value of the landmark frequency on the Van-der-pol equation with the input [Equation (6)]; but what really matters is to cross the unity threshold. The parameters values of the Van-der-pol equation with input [Equation (6)] in our proposed model for the information processing block (what stream) are:


$$\lambda =0.2\;B_{1,...,6}=1.5\;A_{s}=1,\;P_{1,...,6}=4,5,6,7,8,9,\;\omega_{s_{1,...,6}}=4,5,6,7,8,9$$

**Figure 7:**
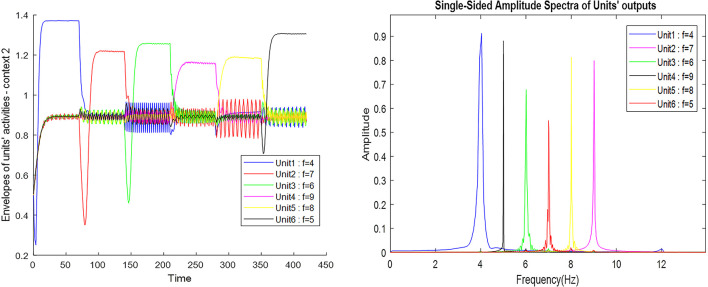
The outputs of the Van-der-pol neuron model proposed in this article to model neuronal ensembles in the PRC region. The simulation results are shown as neuronal units’ responses to input excitations (landmarks 1–6), in the order of encountering based on the route of each context. Left column: amplitude envelope for neuronal units’ responses to input excitations for context 2. Right column: frequency spectrum of neuronal units’ responses to landmarks of context 2. Frequencies 4–9 in this figure are equivalent to main landmarks 1–6 in Figure 3; where their sequential order and consequently the route is different in contexts 1 and 2. The parameters for the Van-der-pol model in this simulation are *λ* = 0.2, *B*_1,‥.,6_ = 1.5, *A*_s_ = 1, *P*_1,‥.,6_ = 4, 7, 6, 9, 8, 5, *ω*_s_1,‥.,6__ = 4, 7, 6, 9, 8, 5.

Figure 7 also shows the frequency and the amplitude of the neuronal ensemble’s activity of the what stream, for a different destination from Figure 3 with a different route. Frequencies 4–9 in Figure 7 are also equivalent to the main landmarks 1–6 in Figure 3.

One of the features of cognitive map based navigation is that an agent can path-plan and navigate from any point of the cognitive map stored in memory as a starting point to any other point of the same map as the destination (not just always from a fixed origin to a fixed destination). With this in mind, the simulation results presented in Figure 7 well demonstrate the capability of the proposed model in *what stream* information processing during navigation in different contexts of the cognitive map.

**Figure 8:**
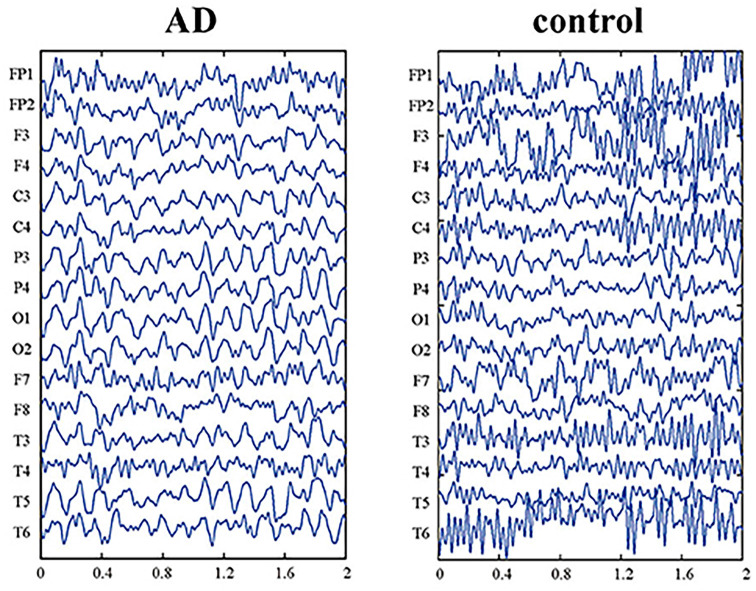
EEG data of Alzheimer’s patient (AD) and normal aging person (control). The picture is reprinted from Yu et al. (2020) with permission.

With the above explanations, in the following paragraphs we explain how the proposed model justifies the disruption in the information retrieval process and, as a result, the disruption in navigation in Alzheimer’s patients. As explained in Equation (4), when the value of the bifurcation parameter, λ, is between zero and one, the neuronal ensemble will oscillate with frequency and amplitude P and 2√λ, respectively. Now, as the value of this parameter increases, we see that other harmonics appear and the main harmonics are pulled to lower powers. In this way, the low-frequency power amplitude increases (Cutsuridis and Moustafa, 2017; Yu et al., 2020). This well equals what happens in the brains of people with Alzheimer’s Disease (AD). According to studies, with the progression of the disease and increased deposition of amyloid plaques in the brain, the high-frequency power of brain activity decreases, and the low-frequency power increases (Figure 8; Zou et al., 2011). Due to the essential and proven role of high frequencies in processes related to memory retrieval, this reduction in high-frequency power and changes in the frequencies’ harmonics, leads to disruption of the memory retrieval process (Lisman, 2005; Traikapi and Konstantinou, 2021). Therefore, we can see that the behavior of the proposed model with an increase in the bifurcation parameter, λ, is equivalent to the result of increased deposition of amyloid plaques in the medial and lateral temporal lobes of the brain in Alzheimer’s patients which causes LM retrieval disruption and navigation disturbance.

**Figure 9:**
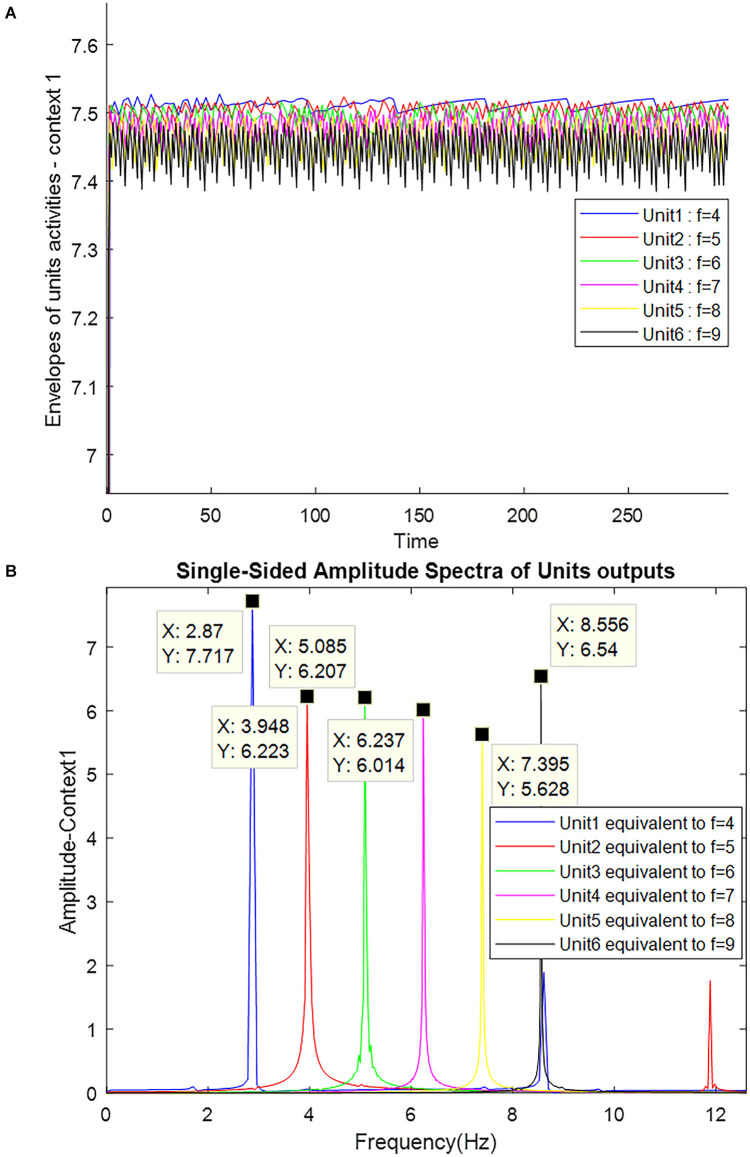
The simulation results of AD patients are shown as neuronal units’ responses to input excitations (landmarks 1–6), in the order of encountering based on the route of context 1. **(A)** Amplitude envelope for neuronal units’ responses to input excitations. **(B)** Frequency spectrum of neuronal units’ responses to landmarks. Frequencies 4–9 in this figure are equivalent to main landmarks 1–6 in Figure 3. The parameters for the Van-der-pol model in this simulation are *λ* = 15.2 *B*_1,‥.,6_ = 1.5 *A*_s_ = 1, *P*_1,‥.,6_ = 4,5,6,7,8,9, *ω*_s_1,‥.,6__ = 4,5,6,7,8,9.

Now, considering ART-based brain function, A consequence of increasing the bifurcation parameter, λ, is that the route landmarks will no longer be recognizable to the AD patient due to the frequency shift of the main harmonics (Figure 9B). As a result, according to what is described in the cognitive model of Figure 1, due to the transmission of the wrong frequency code, the relevant set of rules in the control center (mPFC) will not be activated or another set will be activated. As a result, the AD patient will not be able to remember the landmarks (Figure 9A) and the route and will not be able to make the right decision in choosing the right way. This is the phenomenon of forgetting and losing the path in AD patient. Figure 9 shows the simulations using the proposed model for this case with increased “λ”.

As shown in Figure 9A, none of the neuronal units respond specifically to landmarks. Because the intrinsic frequency of neuronal unit’s activity has changed due to the deposition of amyloid plaques. This change in oscillation frequency can also be seen in the PSD coordinates shown in Figure 9B.

We now focus on describing our proposed model for the mPFC control function during navigation.

### Modeling the mPFC control function during navigation using a fuzzy lookup table system

Choosing the appropriate model for the mPFC has to be undertaken by considering requirements such as the nonlinearity of brain control function over different routes, the possibility of correct routing beginning from different starting points with common or distinct destinations (this indicates the insensitivity of the model output to exact initial conditions, as such sensitivity is observed in non-linear chaotic models), the possibility of considering landmarks in the environment which might also depend on the person’s level of attention, the speed of processing and computational model runtime, which should require a lower amount of computation and higher efficiency (as an attempt to match as much as possible, the actual brain function). In this article, we employ the lookup table based fuzzy system as the mPFC controller model.

Fuzzy logic is in contrast to classical or digital logic which operates on discrete values of either 1 or 0. Fuzzy inference systems can formulate the behavior of a phenomenon or process solely in the form of descriptive and experimental rules without the need to know the exact analytical model. Fuzzy systems design can be done in two ways: using the information of an expert or using experimental data to adjust the parameters of the rule set. In fact, a fuzzy system is a tool for formulating a process using if-then fuzzy rules which form the core of the system. In a fuzzy lookup table system, membership functions are defined to cover the input space using input-output pairs. This means that for any input, there must be at least one set of membership functions in which the membership value of the input is non-zero. If the rule set is not complete for some reason, it can be completed using interpolation. On the other hand, since the equivalent of each input-output pair is a rule, if the number of input-output pairs is greater than the number of membership functions sets, among the rules having the same antecedent, the rule with the highest degree [equivalent to the multiplication of the input (antecedent) and output (consequent) membership value] remains, and others are deleted. The general form of the fuzzy rules is in the form of relation (7):


(7) $$if\;x_{1}is\;A_{1}^{r}\;and\;x_{2}\;is\;A_{2}^{r}...\;and\;x_{N}\;is\;A_{N}^{r}....\;then\;Y^{r}\;is\;B$$

Where r is the rule number, N is the number of input dimensions, A is the input membership function (antecedent), B is the output membership function (consequent), X is the input variable, and Y is the output variable. The membership functions can have different shapes (such as triangular, gaussian, and so on.) and therefore a different number of parameters. The shape chosen for the membership function depends on the number of allowed system-free parameters and the nature of the variables. Also, the system’s inference engine is different according to the nature of the problem. Inference in a fuzzy inference system refers to how the output value is calculated for input data using a rule set. The inference in the fuzzy lookup table system used in this article includes the following steps:

1- Fuzzification
2- Degree of fulfillment or firing strength
3- Implication (use of minimum or multiplier operator)
4- Aggregation (In this part, the outputs of all the rules are combined)
5- Defuzzification

**Figure 10:**
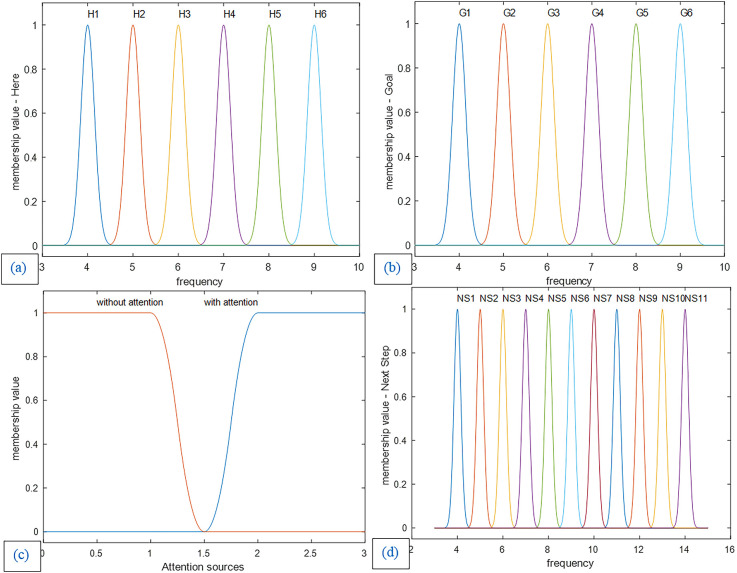
**(a)** Membership functions of the first input: The current location (Here). **(b)** Membership functions of the second input: the goal location (Goal). **(c)** Membership functions of the third input: attention sources. **(d)** Membership functions of the control unit output and decision-making result: Next step. Membership functions of the first and second input and the next step as output are Gaussian with a standard deviation of 0.15, and the membership functions of attention level are smf and zmf.

**Figure 11:**
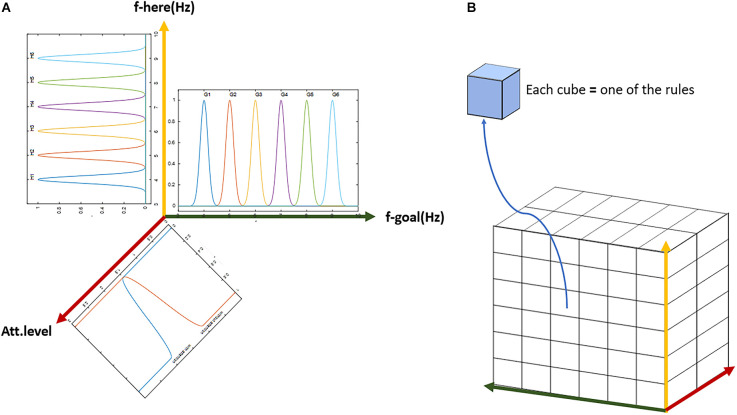
The general scheme of the lookup table based fuzzy system. **(A)** Demonstration of the three-dimensional input with membership functions defined on each dimension. **(B)** The three-dimensional table of rules with complete coverage of the input space. Each block (cube in the figure) represents one rule. The whole set of rules cover all possible options of input and output over the assumed cognitive map with six landmarks and two attention states (with or without).

The fuzzy system design of this article is based on the expert approach. In this article, Gaussian membership functions are used for the current-location landmark and the goal as well as the next step variables (Figure 10). Due to the nature of brain function in sending and processing information based on a synchronous mechanism, our proposed model is based on the frequency of landmarks. Accordingly, in designing our proposed fuzzy lookup table system for the mPFC control role, the identification frequency codes are considered for the environmental landmarks as inputs and all related bottom-up and top-down processes in the brain (This frequency code provides a representative frequency for each of the landmarks and the related actual identification frequency is, in fact, a frequency spectrum with a dominant harmonic that may probably be accessible by recording the brain’s electrical activity). In this article, specific frequency numbers have no role in the nature of the model. So, we have used a separate symbolic frequency as a representative for each landmark. Hence as observed in Figures 10 and 11, the horizontal axis of the “Here” and “Goal” as inputs and “next step” as the output membership functions show the symbolic identification frequency of landmarks. On the other hand, a particular frequency interval is considered for all membership functions to take into account possible changes in the Landmark characteristics and details. For example, if one of the landmarks is the cinema building, the billboard picture changes according to the current movie, but nevertheless, the agent considers it the same cinema building as before. Consequently, using a fuzzy system model results in improved stability of the model output regarding changes in the appearance of the landmarks. No overlap is considered for membership functions because we assume that at any instant, only one landmark for the next step is visible and that all landmarks are independent of each other. For this purpose, the standard deviation of all landmarks is considered to be 0.15. Figures 10 and 11 depict diagrams for the membership functions of the input and output corresponding to the map in 3. Furthermore, in the fuzzy lookup table system employed in this article, the multiplication inference engine and central average defuzzification are used as given in Equation (8):


(8) $$Y_{output}=\frac{\sum_{i=1}^{R}{\bar{Y}}_{i}(\Pi_{j=1}^{N}\mu_{i,j})}{\sum_{i=1}^{R}\left(\Pi_{j=1}^{N}\mu_{i,j}\right)}$$

Where i denotes the rule counter, R the number of rules, j the counter of input dimensions, and N the total number of input dimensions.

As can be seen in Figure 11, along the six landmarks considered in the assumed route, 36 possible options exist for the choice of input-output; and considering two states of attention (with and without attention), 72 rules cover the space of this cognitive map and navigations therein. For the assumed route in Figure 3 and considering two modes for the start and goal points, 16 rules out of a complete set of 72 rules are active in our problem. Tables s 1 and 2 show these 16 rules as used in our simulations.

**Table 1:** Rule set of the lookup table based fuzzy system for Route 1 with the starting point at LM1 and ending at LM6 (as Goal) from the assumed cognitive map in Figure 3.

**Route 1—Start: LM1 Goal: LM6**			
**Here**	**Goal**	**Attention level**	**Next step**
1	6	2	2
1	6	1	7
2	6	2	3
2	6	1	8
3	6	2	4
3	6	1	9
4	6	2	5
4	6	1	10
5	6	2	6
5	6	1	11

**Table 2:** Rule set of the lookup table based fuzzy system for Route 2 with the starting point at LM4 and ending at LM1 from the assumed cognitive map in Figure 3.

**Route 2—Start: LM4 Goal: LM1**			
**Here**	**Goal**	**Attention level**	**Next step**
4	1	2	3
4	1	1	10/5
3	1	2	2
3	1	1	9
2	1	2	1
2	1	1	8

## Results

**Figure 12:**
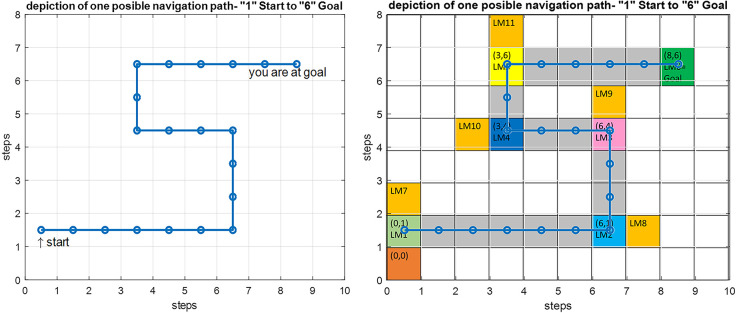
Left: Simulation result for the implemented proposed navigation model in Matlab. Right: An overlay of the simulation output on the predefined route shown in gray with the starting point at LM1 and the goal at LM6.

Figure 12 shows the modeling results for navigation over this article’s assumed route. The shown map is equivalent to the cognitive map stored in a person’s mind. In this route, the agent should move from the start point equivalent to LM1 to the goal equivalent to LM6. In this simulation, the initial values which are used to start navigation are *f(Here)* = 4.2 Hz, *f(Goal)* = 9 Hz, and Attention Level = 2.3. Considering the primary assumption that learning is completed and as a result, the cognitive map and the rules of the control unit have already been formed, and the performance of the agent is computed as follows:

The input data from the where and what streams are processed and converted into the equivalent codes of the cognitive map using Equations (3) and (6). These are then matched against the membership functions of all three input dimensions of each rule. For each input, the antecedent membership values of each rule are collected and multiplied by each other, and according to Equation (8), the weighted average of the rules’ consequent is computed using the average basis values of the output membership functions. Based on this value, the appropriate command is issued by the mPFC control unit for changing or choosing the subsequent path for approaching the next landmark, and this process continues until the goal is reached.

It can be seen that due to the natural stability of the designed fuzzy model against environmental changes (in the allowed range), the process of navigation has been performed correctly, and the person has reached the goal over an optimum path, despite a mismatch at the starting point as compared with the exact symbolic frequencies of the landmarks.

**Figure 13:**
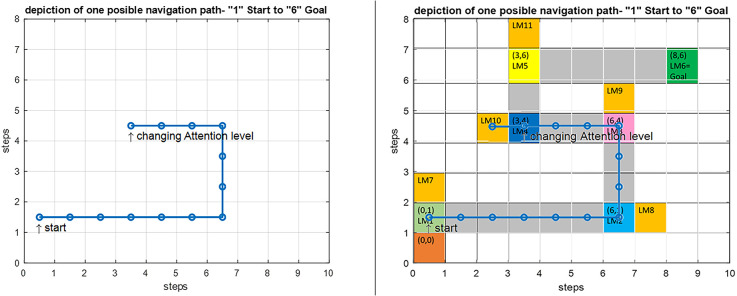
Left: Simulation result for the implemented navigation with attention deficit model in Matlab. Right: An overlay of the simulation output on the predefined route. By introducing a drop in the attention level at LM4, the agent deviates from the route and enters the wrong path denoted by LM10, and stays there until the attention level is raised to correct the routing.

Since attention level as one of the key parameters in the navigation process has been included in our model, several wrong paths are considered in our assumed map such that the effect of attention in our proposed model can be shown. If the attention level is below a specific threshold defined in the model (Figure 10, section c) the agent erroneously enters the wrong path and remains there until the attention level returns to a normal state. These wrong paths have been symbolically depicted by orange-colored blocks and named LM 7–LM11 on the map. Figure 13 simulates a situation in which a person starts navigation from LM1 toward the LM6 as the goal. But this time the attention level of the agent drops in LM4 and the agent deviates from the main path, enters a wrong path which starts with LM10 and will remain there until the attention level returns to the defined threshold.

There are many disorders and diseases in which the attention level control centers and the centers that provide the attention sources (such as dopamine) are disturbed or damaged. In two-way decision points such as LM1–LM5 turning points in Figure 3, if attention deficit causes wrong path planning and navigation, it is assumed that the agent will enter the path with the slightest angle to the previous step direction. But sometimes a disease leads to the destruction and disruption of memory centers and loss of connectivities that support information transfer and memory access in the brain. Navigation disorder in the Alzheimer’s disease is a clear example, where the place cells in the HPC (as the center that stores landmarks and trajectories) are destroyed because of atrophy and as a result cannot respond to the retrieval codes and signals sent by the mPFC control unit. In this case, we assume that the weighted competition of the paths will determine the direction in which the subject continues to navigate. This means that in the agent’s stored cognitive map, at first the landmark and path with the closest code and response frequency to the lost information is retrieved (seems more familiar) and takes on more weight, and the agent will choose the next step and path depending on these weights. With this explanation, if no path seems more familiar and no information is retrieved, chances for all path options are equal, and other factors will determine the direction of the agent’s next step.

## Discussion

To identify the merits and limitations of the proposed model, we shall briefly consider the shortcomings of previous attempts. There are different types of navigation, including beaconing, route following, dead reckoning, and map-based navigation. Our model assumes map-based navigation. During navigation using the cognitive map stored in the memory, the agent can plan a route and navigate from any point of the cognitive map as a starting point to any other point of the same map as a destination (not necessarily from a fixed origin to a fixed destination).

As pointed out in the introduction and background sections, so far several studies have investigated and proposed cognitive and computational models of human navigation in cellular and functional scales. But due to the complexity and multi-faceted nature of this problem, a comprehensive cognitive and computational model of what happens in the brain during this process has not yet been presented. Each study has investigated limited aspects of navigation. In this study, we aim to present a more integrative cognitive and computational model of information retrieval control in the navigation process in accordance with the perceived natural functioning of the brain. In this way, some of the novelties are: considering the interaction between HPC and mPFC, including short-term/working memory in the model, modeling the functioning, receiving, and converting information in Where and What streams, modeling information retrieval control by mPFC, inclusion of the level of attention in the model, providing a model-based justification of what causes forgetting the route in Alzheimer’s patients by analyzing the Van-der-pol model. The presented model agrees well with the behavior of the brain of people with Alzheimer’s disease due to the increase in the deposition of amyloid plaques in the problem of amnesia.

This frequency justification based on ART and the presented more comprehensive cognitive and computational model as compared with previous works, allows researchers to evaluate the effect of pharmacological and non-pharmacological interventions such as fixed and intermittent electrical stimulations in improving the recall and recognition of landmarks and path information, and the general well-being of these patients.

In the following, we will describe and examine the shortcomings of previous models and the achievements of this study in solving some of these shortcomings.

There is strong evidence in the field of brain imaging and electrical activity recording that supports the HPC-mPFC interaction and the essential role of the mPFC in controlling memory retrieval within the HPC during navigation tasks. However, in previous studies, only the simultaneous activity of some areas during a specific task is discussed, or measurement of the cognitive performance of a unit based on the output of a specific experiment is reported (Jeanne Sholl, 2001; Hodgson and Waller, 2006). Also, data traffic between active areas in this process is not explicitly and comprehensively available (Nyberg et al., 2022). In many experiments, the theta signal was observed both during the encoding and the retrieval, but it is still not entirely clear what the nature of the transmitted information is (Wirt and Hyman, 2017), and only hypotheses are presented according to observations from experimental recordings. On the other hand, in the field of modeling, many models have been proposed for path planning and some aspects of navigation, where usually the connections and structures are in the form of black boxes and employ a variety of artificial neural networks (Madl et al., 2015).

One of the drawbacks of these models is that they often do not have a biological correspondence to what happens in the specific brain regions during navigation and are mostly used to guide the movement of robots; also, the matter of active brain regions’ neuronal oscillatory functions during navigation and synchronization of different brain regions in communicating for information transfer, is not reported so far for some key brain regions in navigation models (Droulez and Berthoz, 1991; Byrne et al., 2007; Chersi and Burgess, 2015; Madl et al., 2015).

In an attempt to solve the above-mentioned problems, we proposed a cognitive model incorporating the key brain regions that retrieve the cognitive map during navigation. The resulting model shows a higher degree of agreement with real brain function in navigation. Based on this cognitive model (shown in Figure 1), we further proposed a computational model of navigation with stronger focus on the mPFC control function in cognitive map retrieval during navigation. To model this control function of mPFC, a Fuzzy Lookup Table system has been used. Our proposed rule-based model is very close to the mPFC real function in navigation. Also, the model is robust to changes in some features of landmarks due to the definition of membership functions (and not just a crisp value) for inputs which makes the model stability very similar to the actual brain function during memory retrieval and navigation; since according to daily experiences, despite some changes in the appearance of environmental landmarks, the same route is retrieved and identified in our memory, and these changes do not disrupt the navigation process.

Furthermore, the proposed model provides the facility of modeling and investigating attention-related disorders in navigation, considering the attention level parameter. Likewise, we can study the effect of amnesia on navigation results by removing the information of some paths and landmarks from the cognitive map [equivalent to zeroing their value in the cognitive map matrix of (2)]. This allows us to model patterns of navigation, especially in more complex routes where the agent faces multiple ways at decision points, which help in the early detection of some diseases and disorders associated with wayfinding and navigation problems, including Alzheimer’s disease.

Therefore, the proposed model can be used in cognitive and brain mapping studies and likewise in designing and predicting the outcome of drug interventions and transcranial stimulation in related diseases. As briefly mentioned in Figure 4, a polar network with 360 neurons (optional depending on the desired resolution) is considered to model the receiving and primary processing of the where stream block (parietal lobe and then, PHC). Each neuron is sensitive to the presence of a spatial landmark at a specific distance and angle to the agent and fires when the landmark is placed in such coordinates. For the polar neuron model, RBF neurons can be used, similar to Equation (3).

We also proposed to use the Van-der-pol neuron model with relation (6) to model the synchronous process of receiving and processing what stream information. In this regard, the stimulus in our model is B * sin (ω_LM_t). We used the Van-der-pol model because of its biological justification in Alzheimer’s disease (AD) as one of the most widespread and well-known diseases associated with navigation disorders. For example in AD, due to brain atrophy, it has been observed in brain electrical activity that the spectral power of high frequencies decreases and the power of low frequencies increases (Jensen and Tesche, 2002; Lisman, 2005; Duch, 2007; Zou et al., 2011; Cutsuridis and Moustafa, 2017). This corresponds to an increase in the value of the parameter λ in the Van-der-pol equation. In the Van-der-pol equation, the condition implies periodicity in the behavior of the neuronal population, and the output will be of a frequency nearly equal to P and oscillation amplitude 2$\sqrt{\lambda }$. As λ increases, the amplitude of the oscillation increases and the frequency decreases to less than P. Thus, if we consider the increase in λ to be equivalent to the increase in amyloid plaques and consequently in neuronal ensembles atrophy, both phenomena are justifiable: the spectral power increase at low-frequencies, and the inability to recall the learned landmarks when exposed to them. Because the oscillation frequency of the corresponding neuronal ensemble is no longer close to the frequency of the input signal, so the recognition and retrieval processes are disrupted. Consequently, the relevant set of rules in mPFC is not activated or the wrong rule set is activated. As a result, the process of recall and prediction of the subsequent steps is disrupted and the Alzheimer’s patient will not be able to remember and make the right decision at the crossroads (key landmarks) to choose the right direction and will lose the way. Also, parameter B, which is the coupling coefficient between the input stimulus and the corresponding neuronal ensemble, actually determines the sensitivity of the neuronal ensemble to the input. According to previous studies, the attention control system plays an important role in regulating this sensitivity by regulating the level of neurotransmitters used in attention control areas such as dopamine. The attention control system is also one of the centers that are severely damaged in Alzheimer’s patients. This disorder demonstrates itself by changing the value of parameter B in our proposed model, such that an increase in the value of B results in a corresponding increase in the synchronous speed and reduces the reaction time of the processor units to the input stimulus. From a biological point of view, B can be thought of as a chemical carrier that causes synchronization or desynchronization between neuronal ensembles. So, it cannot continuously increase, and a saturation constraint must be considered.

With these explanations, our proposed cognitive and computational model and the theories embedded therein can be used to extend brain mapping research and to study and predict the effect of pharmacological and non-pharmacological (such as cognitive tasks and extracranial electrical or magnetic stimulation) interventions. Also, according to Wirt and Hyman (2017), the mPFC creates a framework in which it combines various cognitive processes such as emotional valence, rules, and sequences with spatial information, and this framework is ultimately stored in the mPFC. Therefore the proposed model has a firm foundation for such further developments as: (1) adding an emotion unit and its effect on navigation when an agent faces the landmarks; (2) adding the process of learning using a self-organized fuzzy control system; and (3) adding the striatum in the use of procedural memory and stimulation. The special structure of this model, due to the explicitly specified connections of the navigation-related brain regions, and the clear nature of the information transferred between the units, provides a strong potential for the model, which facilitates the completion of the above-mentioned items on the same model.

## Conclusion

This article advances the field of modeling the human brain’s functions in navigation by introducing a cognitive and computational model of navigation with stronger focus on HPC-mPFC mutual interactions and the control function of the mPFC in the spatial memory retrieval from the HPC during navigation based on the concept of synchrony. For this purpose, we used polar neuron and Van-der-pol neuron models to model *what* and *where streams*, respectively. Then, due to the nature of mPFC performance based on rules (the stored memory of previous attempts and their outcomes) during the spatial memory retrieval, we used a fuzzy lookup table system model. Furthermore, we presented a justification for the forgetting of landmarks and routes in AD patients based on changes in the Van-der-pol model bifurcation parameter and in agreement with neuro-biological findings. Also, the model output was investigated for navigation on the assumed route under healthy and attention-deficit conditions. Analysis of the results demonstrates the model’s capability and promising applicability in extending brain mapping research and predicting the effects of pharmacological and non-pharmacological interventions. The model can be extended in several respects, such as modeling the effect of emotion on navigation upon encountering the landmarks, and an addition of the process of learning and cognitive map formation.

## Data Availability Statement

The original contributions presented in the study are included in the article, further inquiries can be directed to the corresponding author.

## Author Contributions

MM performed the literature review, produced the main ideas, and did the modeling, computation and analysis and also wrote the manuscript. FT and SG have provided supervision and effective scientific advice and related ideas, research design guidance, and added value to the article through editing and contributing completions. All authors contributed to the article and approved the submitted version.
